# Cyber and Physical Security Vulnerability Assessment for IoT-Based Smart Homes

**DOI:** 10.3390/s18030817

**Published:** 2018-03-08

**Authors:** Bako Ali, Ali Ismail Awad

**Affiliations:** 1 Department of Computer Science, Electrical and Space Engineering, Luleå University of Technology, 971 87 Luleå, Sweden; bakoali@hotmail.com; 2 Faculty of Engineering, Al Azhar University, P.O. Box 83513 Qena, Egypt

**Keywords:** smart homes, the Internet of Things (IoT), security risk assessment, OCTAVE Allegro

## Abstract

The Internet of Things (IoT) is an emerging paradigm focusing on the connection of devices, objects, or “things” to each other, to the Internet, and to users. IoT technology is anticipated to become an essential requirement in the development of smart homes, as it offers convenience and efficiency to home residents so that they can achieve better quality of life. Application of the IoT model to smart homes, by connecting objects to the Internet, poses new security and privacy challenges in terms of the confidentiality, authenticity, and integrity of the data sensed, collected, and exchanged by the IoT objects. These challenges make smart homes extremely vulnerable to different types of security attacks, resulting in IoT-based smart homes being insecure. Therefore, it is necessary to identify the possible security risks to develop a complete picture of the security status of smart homes. This article applies the operationally critical threat, asset, and vulnerability evaluation (OCTAVE) methodology, known as OCTAVE Allegro, to assess the security risks of smart homes. The OCTAVE Allegro method focuses on information assets and considers different information containers such as databases, physical papers, and humans. The key goals of this study are to highlight the various security vulnerabilities of IoT-based smart homes, to present the risks on home inhabitants, and to propose approaches to mitigating the identified risks. The research findings can be used as a foundation for improving the security requirements of IoT-based smart homes.

## 1. Introduction

**Figure 1:**
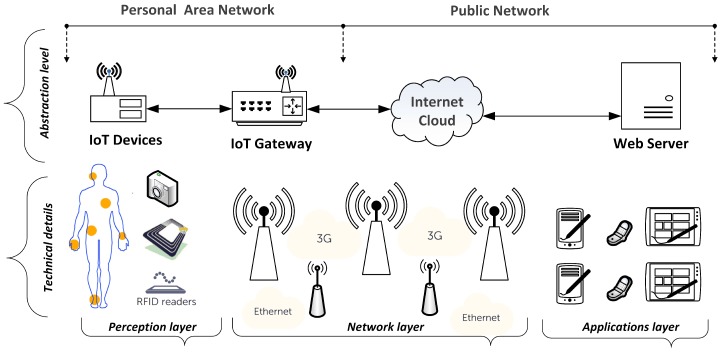
A generic architecture of an IoT system comprises IoT devices, a gateway, and a web server. The figure shows the internal and external sides of the system. The figure was modified from [1].

The Internet of Things (IoT) is an emerging paradigm due to extensive developments in information and communication technology (ICT). The inclusive IoT infrastructure contains a network of devices or objects such as embedded computers, controllable sensors, and radio frequency identification (RFID) tags, in addition to the IoT gateway and the remote server [1]. The architecture of a common IoT system is divided into three layers: perception layer, network layer, and applications layer. The way in which components are grouped together in the three layers of a generic IoT system is shown in Figure 1. From another viewpoint, the term “things” in the IoT model comprises both the cyber world (entities, cyber actions, cyber events, and services) and the physical world (objects, behaviors, tendencies, and physical events) [2].

The purpose of IoT is to expand the functions of the first version of the Internet by increasing the ability to connect numerous objects. By using the IoT model, users can share both the information provided by user behavior and the information collected by the connected things in the physical world [2]. The IoT deployment process involves different technologies, such as wireless sensor networks (WSNs), RFID, Bluetooth, near field communication (NFC), internet protocol (IP), electronic product code (EPC), wireless fidelity (Wi-Fi), sensors, and actuators [3,4]. The key objective of the IoT paradigm is to enable users to uniquely identify, signify, access, and control things at anytime and anywhere via the Internet [1,5]. Interconnected device networks can produce numerous intelligent and autonomous applications and services that offer personal and economic benefits to society [6].

Although several definitions of smart homes exist, from the technical viewpoint, the common concept is to connect sensors, home appliances, and smart devices via the Internet to achieve remote monitoring of, remote access to, and remote control of a residential environment [7]. Therefore, smart environments target rich combinations of small computational facilities to identify and deliver personalized services to users who interact and exchange information with the environment [5]. A smart home can also be defined as a home that is automated, via the application of the IoT paradigm, and capable of reacting to the requirements of its inhabitants, providing comfort and security [8].

From the social perspective, the smart home environment is referred to as ambient intelligence, which is sensitive and adaptive to modern human and social needs [9]. The IoT application domains are very important and will increase over time, as they offer powerful means to help and support the special needs of the elderly and people with disabilities [10], enabling users to monitor and control the environment [11]. The primary objectives of a smart home are to increase home automation, simplify energy management, and reduce environmental releases [12]. Furthermore, the energy consumption level and residents’ comfort are key factors when designing any smart home environment [13].

**Figure 2:**
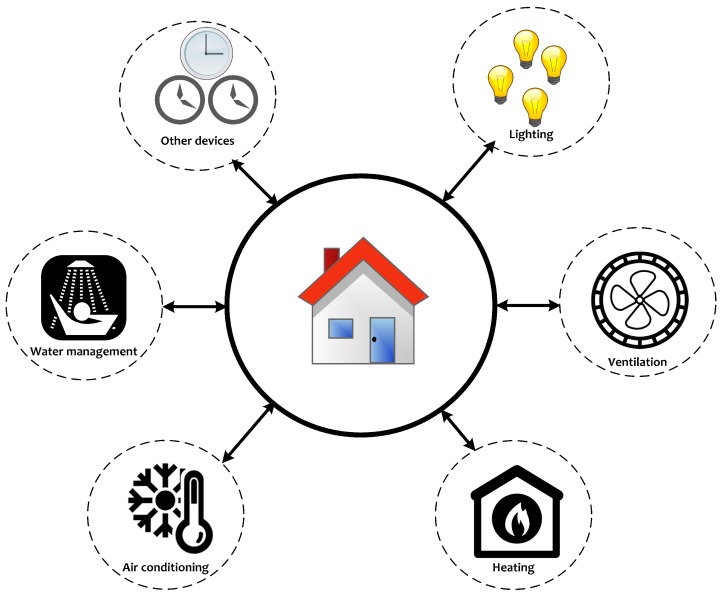
Examples of some controlled environmental services in smart home environments.

**Figure 3:**
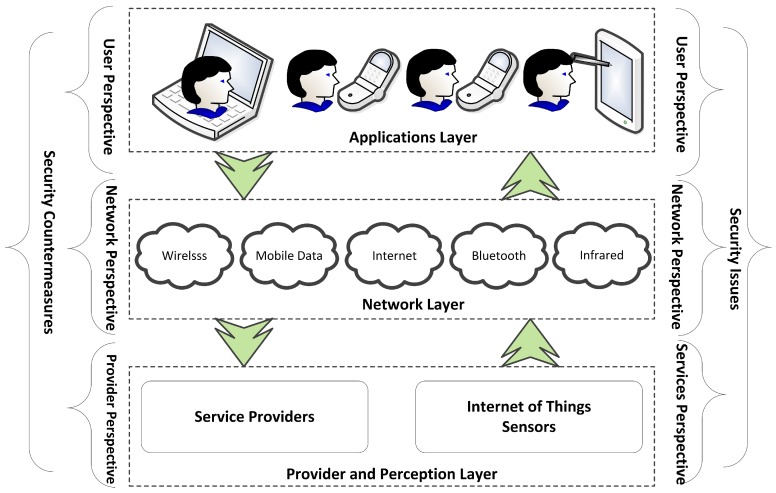
An IoT system from provider, network, and user perspectives. The figure highlights the points of weakness of the systems corresponding to the IoT layers.

A smart home focuses on the automation and control of environmental services such as day lighting, heating, ventilation and air conditioning systems [14], monitoring and control, safety and security, and energy savings [15]. An example of the controlled environmental services of a smart home is shown in Figure 2. Technically, a home automation system consists of five building blocks: devices under control, sensors and actuators, the control network, the controller, and the remote control devices [16]. A complete picture of the IoT components, service providers, different IoT layers and their possible security issues is presented in Figure 3. From the offered services point of view, different kinds of smart home services exist, such as smart homes for security, smart homes for healthcare, smart homes for eldercare, smart homes for childcare, and smart homes for energy efficiency [17].

Home automation systems can be split into two main categories: locally controlled or remotely controlled systems. Locally controlled systems utilize an in-premises controller to carry out an automation process. These systems offer inhabitants a complete use of a home’s systems from within their home via a stationary or wireless interface [18]. Remotely controlled automation systems use an Internet connection to offer users a full control from their personal computer or mobile device. The system can work via integration with an existing home security system and can be controlled using a smartphone via a home security provider [19].

The deployment of IoT technology for building smart homes, with respect to automation and control processes, presents new security challenges. Thus, IoT-based smart homes require a new level of security requirements, as the smart home environment will contain important, sensitive, and private information. Since IoT technology offers opportunities and imposes risks, an IoT-based smart home is susceptible to the IoT security vulnerabilities and is highly vulnerable to attacks via the Internet. If a smart home or a smart device is hacked, the adversary has the potential to invade a user’s privacy, steal personal information, and monitor users inside a smart home environment [20].

The number of IoT devices has rapidly increased, with a recent estimate suggesting that there were 12.5 billion Internet-attached devices in 2010, which is predicted to increase to 50 billion devices by 2020 [21]. By bringing IoT technology into our homes, there are trade-offs between convenience, control, security and privacy. Privacy and security issues should be treated with a high degree of flexibility, as advocated in [22,23]. Therefore, security is one of the areas that must give the highest priority when implementing smart home technology.

This article tackles the problem of IoT-based smart home security risks. The research contributions are threefold: first, this research applies the operationally critical threat, asset, and vulnerability evaluation (OCTAVE) methodology known as the OCTAVE Allegro risk assessment methodology to identify security risks originating from inside and outside smart homes. Second, it considers a holistic view of both cyber and physical security risks within the IoT-based smart home domain. Third, the study proposes several countermeasures for mitigating the identified security risks. These contributions should enhance the existing security policies for IoT-based smart homes.

The rest of this paper is structured as follows: Section 2 provides information on the work related to this study. In Section 3, the OCTAVE Allegro methodology is explained and connected to the addressed problem. Section 4 presents the research findings in terms of possible threats, identified risks, and proposed mitigation approaches. A comprehensive discussion on the research findings is given in Section 5. Finally, conclusions and planned future work are provided in Section 6.

## 2. Related Work

Wireless smart sensors have become very attractive devices for monitoring and tracking moving objects in smart home applications; therefore, they have become a target of different attacks. There are different attacks on WSNs, such as those related to (1) services availability (flooding, jamming, and replay attacks), (2) network routing (unauthorized routing update and wormhole attacks), and (3) node authentication (eavesdropping and impersonation attacks) [24].

Although many benefits are gained from IoT-based smart homes, these smart homes are susceptible to different attacks [25]. An individual can directly attack an interconnection device (e.g., gateway) or field device using its network or local communication interface (i.e., attacking the device) [14], and a device can be impersonated using its faulty certificate. Household appliances can be connected to a wired or wireless network via the home gateway. An attack against the home gateway can immediately lead to an attack against the whole household network, as it is the point at which an outside connection can be made [26,27,28].

It is necessary to protect smart homes against attacks, at both the backbone level and the control level, originating from both the outside and the inside of a smart home. An attack can occur at the traffic level, the control level, or the backbone network level. A direct attack on a device interconnection point (e.g., gateway) or a field device can be carried out using its network or local communication interface. As an example, electricity pricing manipulation may result in reduction of the adversary’s bill at the expense of the user (i.e., the user’s bill is increased). The study in [29] proposed a technique that can be used to effectively detect electricity pricing manipulation.

From the IoT hardware viewpoint, IoT devices are mobile and may arrive in a given smart environment from an unknown domain. The problem is that even a known device may have been altered during its absence [30]. The types of security vulnerabilities include hacking of the home device, a virus attack, an information leak, content fabrication and privacy violation. There are various ways to infiltrate smart homes. Depending on the intentions of the adversary, different groups of smart home devices will be of interest. The first widespread attacks will most likely target products of the controlling system group, as they are most similar to existing targets and are connected to almost every other smart home device [31].

The study in [28] concluded that an adversary has two different opportunities to obtain access to control functions: network attacks and device attacks. In network attacks, an adversary may try to intercept, manipulate, fabricate, or interrupt the transmitted data. Device attacks can be classified into software attacks, physical or invasive attacks, and side-channel attacks. Additionally, there is the possibility that an attacker can disguise itself as an internal user via an interactive digital TV or access a TV illegally via other means to control the home appliances.

In [32], the authors described the types of attacks typically aimed at WSNs and an intrusion detection system that can be used to prevent against them. The authors described cyber attacks that occur in wireless sensor networks, namely, denial-of-service (DoS) attacks, misdirection, selective forwarding, sinkhole attacks, Sybil attacks, wormhole attacks and HELLO flood attacks. Privacy and tracking are the two most important security questions that arise from RFID technology, though there are some others worth mentioning, such as physical attacks, DoS attacks, counterfeiting, spoofing attacks, eavesdropping, and real-time network traffic interception, collection, and analysis [30,33,34].

In [35], the authors presented a smart home risk analysis using the information security risk analysis (ISRA) method. The System’s risk exposure was reviewed with respect to the confidentiality, integrity, and availability. The analysis process was conducted using empirical information gathered in a software development phase. The risk analysis considered five system components, namely, sensors in smart homes, cloud servers, in-house gateway, smart phone apps, and application programming interfaces (APIs). The identified risks were grouped into five categories, namely, software, hardware, information, communication, and human-related risks. A total of 32 risks were examined: nine risks were classified as low, four risks were classified as high, and the rest were classified as moderate. While the study in [35] focused on cyber risks, the work done in this research considers a holistic view of smart home environments by identifying cyber and physical security vulnerabilities using OCTAVE Allegro methodology.

Several risk assessment studies are available in the literature, such as [36,37,38]. However, these studies emphasize risks to general IoT systems and are independent of the IoT application domains. In general, risk assessments designed for an IoT architecture can cover the three IoT layers, but it is not necessary for these studies to cover the security risks in smart homes due to the lack of user behavior and physical security considerations in the context of IoT-based smart homes.

To achieve better safety and security for remotely monitored and controlled systems, the study in [39] proposed a phone-out-only policy and a virtual environment strategy. The aim of the phone-out-only policy was to ensure that the communications between the smart home devices and the remote users are initiated by the smart home devices from the indoor side only. The proposed system enabled a user to easily monitor and control a microwave oven, a security camera, the central heating system, and the washing machine from anywhere by using mobile phones. The work done in [40] recognized the major attacks on the smart home environment: (1) eavesdropping, (2) DoS attacks, (3) information hijacking, and (4) sinkhole and wormhole attacks.

The study in [41] presented a security model for protecting the information flow in the home area network of a smart grid. The proposed model was able to effectively manage the information flow in a home area network using the confidential and non-confidential information flow policies without affecting the normal home area network functionality.

In short, the identified studies on smart homes provided above focused mainly on possible security issues that may occur in smart environments. There is no research that covers the entire IoT architecture from both the cyber and physical perspectives. However, the identified studies focused on either the IoT paradigm or some parts of smart home systems. This study takes a step further by carrying out a security risk assessment of IoT-enabled smart homes, taking into account both cyber and physical viewpoints.

## 3. Risk Assessment Approach

The methodology adopted for this research is the OCTAVE Allegro methodology [42]. The risk assessment methodology was selected to allow comprehensive risk assessment, yielding robust results, and focuses mainly on information assets. The OCTAVE Allegro approach analyzes how the information is used by the users or devices in a system. In addition, it considers the information containers as the locations where the information exists and how this information is exposed to risks. Other critical assets can be identified and assessed by developing connections to the initially identified information asset.

**Figure 4:**
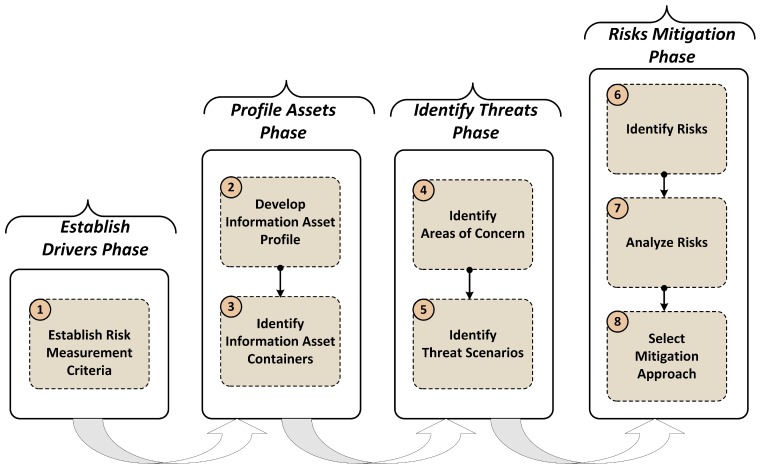
OCTAVE Allegro methodology flowchart of the eight steps, which are categorized into four major groups. The figure was excerpted and modified from [42].

This study focuses mainly on information asset security and on where that information exists when conducting the security risk assessment of a smart home environment. Almost all important assets can be easily assessed and processed using information containers. OCTAVE Allegro provides guidance, worksheets, and questionnaires for conducting the risk assessment process. OCTAVE Allegro is well suited for the risk assessment of smart homes due to the possibility of having an asset container that covers both cyber and physical security. The utilized method has eight steps grouped into four major phases, as shown in Figure 4 [42,43].

### 3.1. Establish Drivers Phase

The goal of the establish drivers phase is to create a foundation for the information asset risk assessment by developing a set of risk measurement criteria for a smart home. These criteria offer the ability to measure the extent to which smart home stakeholders are affected in the event of an information asset breach. Beyond recognizing the extent of impact, the most significant impact area needs to be determined. These criteria reflect a range of impact areas that are important to smart home residents. For instance, impact areas can include the health and safety of users, finances, reputation, and laws and regulations.

### 3.2. Profile Assets Phase

During the profile assets phase, which includes step 2 and step 3 shown in Figure 4, critical information assets are initially identified and then profiled. In the profiling process, clear boundaries for an asset are established, and the security requirements are identified. Afterwards, all locations at which an asset is stored, transported, or processed are determined. In addition, where these assets are used by smart home owners or smart home systems, how these assets are accessed, and who is responsible for these assets should be determined. Logical, technical, physical, and people assets are documented. In this way, the points of weakness at which the security requirements, in terms of the confidentiality, integrity, and availability (CIA) triad, of the information asset can be compromised are identified.

### 3.3. Identify Threats Phase

In this phase, which includes step 4 and step 5, the focus is on the identification of the security threats from the identified assets in the context of the locations at which the information asset is stored, transported, or processed. The security vulnerabilities or the areas of concern are determined and expanded into threat scenarios that further form the properties of the threat. Finally, specific threats that can negatively affect asset security are highlighted.

### 3.4. Risk Mitigation Phase

In the risk mitigation phase, which includes step 6, step 7, and step 8 shown in Figure 4, the cyber and physical security risks in terms of the information assets are identified by determining how the threat scenarios can impact a smart home system. The assessment are carried out via analysis of the impacts or consequences of those threats on the smart home environment. Finally, a mitigation strategy is determined for each of the identified risks. The risks are analyzed and assigned a qualitative value to describe the extent of impact on smart home users. The impact value is derived from the risk measurement criteria, and the scoring information is used to rank the identifier risks and prioritize the proposed mitigation actions.

## 4. Results

The aim of this section is to first collect all security threats found by conducting the information security risk assessment using the OCTAVE Allegro methodology. The results of this research are presented in Table 1 and Table 2, which give a better overview of the identified security threats and the potential risks in an IoT-based smart home environment. The two tables show the information assets that were identified and used in the risk assessment process, the threats associated with them, and the consequences or potential impacts in the form of specific risks and risk scores.

Table 1 shows the threats identified as a result of studying the entire IoT-based smart home system in terms of the cyber and physical perspectives and according to different assets containers. The identified risks cover the user authentication, user behavior, smart home devices, and data exchange among home devices via the Internet. In Table 2, the possible impacts or potential risks are determined and connected to the assets and threats mentioned in Table 1.

In the impersonation of a legitimate home user threat in Table 1 (Threat ID 1), an adversary tries to impersonate and act on behalf of a legitimate home user. To achieve the adversary’s goal, access to the inhabitants’ credentials usually consists of a User ID, and a Password is required. The access to user credentials can be done via social engineering or by intercepting plain data that provides access to the IoT resources. Social engineering can be explained as an approach to deceive or influence people to disclose sensitive information [44]. A malicious code can be injected into applications installed on the IoT system, which makes it possible for attackers to execute harmful operations. The malicious code injection threat is assigned to (Threat ID 2), and it is also connected to user impersonation in (Threat ID 1).

Device compromising threats in Table 1 (Threat ID 3) may lead to situations where sensors are not able to detect physical risks such as fire, flood, or any strange movement within a home. In addition, by stealing information collected by the installed sensors as mentioned in (Threat ID 6), an attacker can inject a malicious code, a virus, or a worm into network traffic and then release it in the system or the mobile applications. Intensively using system’s resources via constant self-replication, resulting in the system being unable to complete relevant work and bringing the system down, eventually making the smart home system completely unusable.

By gaining access to location data from mobile or GPS-enabled devices as reported in (Threat ID 9), an adversary person may conclude that a smart home’s resident is not at home, which may lead to more serious consequences such as financial loss due to home robbery. Table 3 gives further details and real-world examples related to the possible security risks mentioned in Table 2.

Possible countermeasures with the goal of protecting information assets, and hence making a smart home more secure, are reported in Table 4. The key concepts of the proposed mitigation approaches are correct technical configurations, strong user authentication, and home resident security awareness. The proposed countermeasures are correlated with security threats and risks.

Using a strong authentication method such as biometric identifiers is the first proposed countermeasure in Table 4 (Threat ID 1). Biometric traits include, for example, fingerprints, hand geometry, retinal scan and iris patterns, and signature. In addition to its strong authentication capability for civilian and forensic applications, biometric is considered as a non-intrusive approach suitable for people with mental disorders who can not remember their credentials [45,46]. It also offers a good possibility to be implemented on hardware platforms [47]. The best way to keep security on users’ attention is to offer continuous security awareness and education programs [48]. Multi-factor authentication is the process of identifying a user by validating two or more claims presented by the user, each from a different category of factors that include something you know, something you have, or and something you are.

Using secure Wi-Fi connections within smart home environments, (Threat ID 2), stops an adversary from hijacking the network link, and, hence, reduces the possibility to access sensitive data by sniffing the network traffic passing through or injecting malicious codes into the system [28]. Hijacking a wireless connection creates a vulnerability where an attacker can inject harmful code that can be executed by some mobile applications. Adversaries can use off-the-shelf tools like WebView API to embed Web-based contents into mobile applications [52].

In Table 4 (Threat ID 3), by using secure communication channels, limiting traffic access to only authorized users, and conducting security training, information modification, information disclosure, and device or sensor compromising can be avoided. This should reduce the potential risks of device manipulation, and, hence, reduce financial losses. The same scenario can be executed for (Threat ID 6). A continuous examination of network traffic, securing of access to system configurations, and the monitoring of system behavior should prevent stealing information through smart home networks. In turn, this should reduce system’s downtime, reduce the possibility of exhausting system’s resources, and alleviate the possibility of new security vulnerabilities being injected into the smart home system.

Performing frequent data backup and archiving, (Threat ID 4), keeps copies of sensitive data and protects them from both physically and technically damage. Securing the backup media should be ensured by having and applying security policy, assigning backup software access rights only to authorized persons, storing the backups off-site, controlling the physical access where the backups are stored, using a fireproof and media-rated safe, and using password-protected and encrypted backups.

Securing the physical location and the access to the devices configuration interfaces should be taken into account. The proposed mitigation approach, (Threat ID 5), represents this issue and recommends physical security considerations. If a biometric authentication approach is going to be integrated to the smart home system, the same approach can be used for applying logical and physical security constrains. Applying the same authentication mechanism on for logical and physical access control should increase the smart home system’s cost effectiveness.

It is worth mentioning that the proposed mitigation approaches do not offer full solutions to the identified threats and risks; instead, the countermeasures are regarded as methods to curb security threats and reduce security risks and consequences. The strict deployment of security countermeasures will result in a decrease in system usability. Again, in Table 4 (Threat ID 9), completely disabling the location tracking service can protect part of the user’s privacy, but it may drastically reduce the device’s usability. Therefore, a good balance between system security and usability should be sought out. A security and privacy improvement framework for mobile devices like the proposed one in [53] can be considered as part of the mitigation approaches.

Choosing authentic providers of IoT devices and system’s components is part of the mitigation approaches, mentioned in Table 4 (Threat ID 10). The IoT devices bought from predatory providers may contain harmful codes or wrong configurations that may compromise any implemented security constraints. Furthermore, to keep the system’s operations and sustainability, regular maintenance, configurations checks, and error rectification should be carried out by authentic and well-trained personnel, and according to legal contracts.

The application of the OCTAVE Allegro methodology requires the utilization of different worksheets for risk identification and mitigation. Including in this research all the worksheets that have been developed is a difficult task due to space limitations. However, further details regarding the worksheets and the risk assessment process are documented in [54].

### Risks and Mitigation Approaches in Action

In relation to Figure 2, a typical IoT-based smart home environment includes a wide variety of devices, services, and vendors. Smart home devices and their suppliers can be divided into six categories, namely, electrical power distributions, smart home controllers, building applications, home appliances, communication devices, and IT and telecom providers [55]. This section maps some of the identified risks and mitigation approaches to a typical smart home environment.

**Figure 5:**
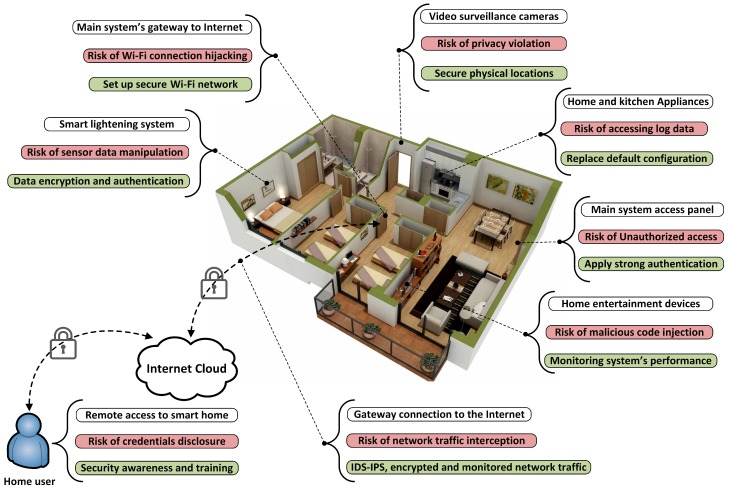
Security risks and mitigation approaches are pointed to an actual smart home environment. The floor plan was borrowed from Amazing Architecture [57].

IoT-based smart home architecture is divided into three layers; device or perception layer, network layer, and applications layer. Figure 5 represents a typical IoT-based smart home environment, and shows the identified security risks and their corresponding countermeasures mapped to the smart home environment. In reality, security risks can cross more than one IoT layer. For example, the risk of unauthorized access can be found in accessing the main system configurations, accessing the IoT gateway, and in the login to the smart home applications. Therefore, a strong authentication method needs to be implemented in all of these points. Biometrics technology can be embedded in multi-factor authentication to build a strong user authentication mechanism [56].

IoT devices installed in smart homes lack high computational power, large storage space, and large memory size. Therefore, implementing intensive security solutions may not be an available option. To offer a secure connection between the IoT devices and the gateway inside the smart home environment, a distributed encryption mechanism [1] or an energy-efficient data encryption approached like a triangle based security algorithm that uses efficient key generation [58] should be taken into consideration.

At the edge of network layer, the IoT gateway works as an intermediary between IoT devices and the external network. The IoT gateway is susceptible to different security exploits such as man-in-the-middle attack and possibility to collect data from IoT devices. Therefore, gateway security is a crucial need for protecting the data flow inside and outside smart home environments. A secure gateway can be built by implementing efficient security algorithms like elliptic curve cryptography (ECC) [59] and using strong user authentication approaches [60].

To achieve a high security level on the entire data path, from IoT device to the home user on the remote side, the network connection to the Internet service provider (ISP) should be secured. Common network security mechanisms such as virtual private networks (VPNs) should be implemented for providing an encrypted link to the ISP. A distributed intrusion detection system (IDS) for IoT networks has to be deployed [61]. Furthermore, traffic collection and monitoring using commodity hardware and software can be deployed to build an early-warning system for detecting any abnormal behavior in the network traffic [33,34].

## 5. Discussion

IoT-based smart homes are highly vulnerable to attacks via the Internet. If the entire smart home system or a smart device is compromised, the adversary will be able to invade the privacy of smart home inhabitants, steal personal or sensitive information, control the smart home system, and even monitor residents inside the smart home environment. Pursuing security risk assessment is the initial step towards understanding smart home security, enabling the identification of emerging security vulnerabilities and hence facilitating the establishment of appropriate security requirements.

This study has conducted a comprehensive security risk assessment using OCTAVE Allegro and identified 10 critical information assets. Via a risk assessment, approximately 15 security risks from the cyber and physical perspectives, as reported in Table 1, originating from both inside and outside smart homes, have been identified. Intuitively, there are other risks that have not been stated due to time limitations and the increased number of worksheets in the OCTAVE Allegro method. The impacts of security risks are described in Table 2. Suitable countermeasures for mitigating the risks to an acceptable level (since 100% security is never attainable) are proposed in Table 4.

The risk assessment aims to identify the most severe potential dangers with a given risk score, as shown in Table 2. Physical security risks correspond to devices and sensors, i.e., the perception layer in Figure 1 and Figure 3. The risks to hardware concern the theft, defect, manipulation, and sabotage of the various devices inside or outside a smart home environment. The highest risk score, which is 41, is related to cyber or information assets such as user credentials, mobile personal data, and user applications. Within network communication, represented by the network layer in Figure 1 and Figure 3, the core risks come from inadequate authentication mechanisms, a lack of secure communication channels, and a lack of appropriate data encryption mechanisms [62].

Reliable user authentication methods such as biometrics should be considered and applied to IoT-based smart homes. Biometrics science aims to achieve human identification or verification based on physiological or behavioral characteristics. Biometrics has been widely utilized in both civilian and forensic applications [45]. Due to its accuracy and reliability, fingerprints as a biometric trait can offer a strong security level for cyber and physical access in a short processing time [63,64]. Biometrics has also applications in e-health security [46] that can be utilized for smart home wearable devices. Authentication protocols for WSNs and the Internet of Things should be considered as well [65].

Although this study proposed precautions for mitigating security risks, the precautions consider the end-user side. Device manufacturers and application programmers should also work towards supplying devices with more security capabilities and applications with secure and easy-to-configure user interfaces. Governmental authorities need to become more involved by offering legal support, security standards, and law enforcement policies.

The proposed mitigation approaches should be used to reduce the security threats and hence to alleviate the potential risks. Increasing system security by applying more security solutions will impact the overall system usability. Therefore, when using some of the proposed countermeasures, both system security and usability should be balanced. In addition, other factors that influence smart home system security, such as an inhabitant’s education level and security awareness, should be taken into account when employing security countermeasures in IoT-based smart home systems.

The risk assessment outcomes demonstrate that human factors are the greatest causes of risks, as not only security administrators, but also people of all ages with different technical backgrounds can live in smart homes, which represent the highest risk exposure. Smart home residents with limited technical knowledge are more vulnerable to social engineering attacks and to system’s misuse and misconfiguration; thus, a security awareness program is a must in all cases to reduce the number of security risks and the amount of anticipated damage.

The research findings and the proposed mitigation approaches can enable all stakeholders, especially end users, to be aware about various security risks and to take appropriate security mitigation measures to improve security in IoT-based smart homes. Furthermore, the research findings establish useful contributions that can be used as a foundation for updating the security requirements in IoT-based smart homes and for improving the existing security policies.

## 6. Conclusions

Applying IoT technology to smart homes yields both opportunities and security risks. IoT-based smart homes are highly vulnerable to different security threats from both inside and outside the home. If smart home or smart device security is compromised, the user’s privacy, personal information and even safety will be at risk. Therefore, appropriate measures have to be taken to make smart homes more secure and suitable to live in. A careful assessment of security risks must precede any security implementation to ensure that all the relevant, underlying problems are first discovered. This paper has successfully conducted a comprehensive security risk assessment using the OCTAVE Allegro method and has identified 10 critical cyber and physical assets. As a research outcome, approximately 15 security risks originating from both inside and outside smart homes have been identified. The consequences or impacts of these risks have been described, assuming that the threats are realized. Suitable countermeasures for mitigating the risks to an acceptable level have been proposed. The focus of this research has solely been on the identification of security threats, impacts or risks, and suitable countermeasures for IoT-based smart homes. The complexity of smart services was not within the scope of this research; thus, no smart home system was built. Our future work will be to develop a framework for realizing and evaluating security risks within the IoT-based smart homes.

## Figures and Tables

**Table 1:** Security threats found by performing an information risk assessment in terms of the possible threats associated with information assets.

Asset ID	Information Asset	Possible Security Threats
1	User credentials	User impersonation
		Identity and credential theft
2	Mobile personal data and apps	Malicious code injected into apps installed on a phone
3		Information modification
	Information collected by devices	Denial-of-service (DoS) attacks
		Device or sensor compromising
	Smart home status information	Information disclosure
		Function interruption
4	Smart home structure	Gain access to inventory information to search for a specific device with known vulnerabilities to attack smart homes
	Inventory information	
5	Log information	Gain access to log data and obtain useful information enabling possible attacks on a smart home system
6	Information transmitted via a gateway	Steal information from packets transmitted via a gateway
7	Smart home setup information	Information modification
8	Video feed of surveillance cameras	Control cameras to monitor and spy on users
9	Location tracking information	Observation of location data traffic
10	Information resources (e.g., pictures, documents, and music)	Steal private information
		Make stored media inaccessible due to hardware failure

**Table 2:** Security risks identified by performing the information risk assessment in terms of the possible impacts and the risk score.

Threat ID	Possible Impacts (Risks)	Risk Scores
	Unauthorized access to the main smart home system	
1	Unauthorized execution of operations	41
	Loss of control over smart home system	
	Adversary can take photos, record conversations, and track locations	
2	Attacker can control the smart phone remotely	41
	Attacker can make calls and access the phone microphone and camera	
	Sensor measurements are manipulated to infiltrate the home system	
3	Non-presence tracking leads to home break-in	39
	Financial losses	
	Attacker identifies the weakest device with known vulnerabilities	
4	Attacker takes control of smart home systems	39
	Financial losses	
	Attacker finds a way to access the main system	
5	Attacker changes the system configuration and adding back doors	39
	Financial losses	
	System resources are exhausted via constant self-replication	
6	Possibility of bringing the system down, making it ultimately unusable	39
	Possibility of injecting new security vulnerabilities into the system	
	Difficulty in setting up the smart home system correctly	
7	Misuse of SH systems with the possibility of malfunction	36
	Financial losses	
	User privacy violation	
8		34
	Financial losses	
	User privacy violation	
9	Breaking into the smart home if it is vacant	34
	Financial losses	
	User privacy violation	
10	Loss of information	23
	Damage to reputation	

**Table 3:** Real-world examples related to the identified security threats and risks from different information assets.

Asset ID	Real-World Examples
1	An unauthorized individual obtains the necessary credentials and is able to login into the main smart home system.
2	The legitimate user loses his or her mobile device or it becomes stolen, and then the smart home-related apps are manipulated. The phone application can be manipulated remotely via injecting a malicious code.
3	An information asset is altered intentionally by malicious individuals to cause the power supply smart meter to show high electricity consumption.
	Jamming and tampering at the physical layer could prevent sensors from detecting risks such as fire, flood, and unexpected motion.
	A compromised motion sensor could be used to determine when there are people at home.
	The statuses of door locks and alarm systems could be used to determine when a smart home is occupied.
4	Attackers can gain access to this information asset by obtaining unencrypted backup media or via a social engineering attack.
5	This asset can be obtained if the log data are easily accessible via an insecure channel.
6	This asset can be obtained if the gateway is not properly secured, e.g., an open Wi-Fi network. The adversary can hijack the Wi-Fi connection, can inject a malicious code, and then takes control over the smart home system.
7	This asset can be obtained if the information asset is stored as a data file in the smart home system (e.g., a PC) without strong authentication mechanisms.
8	This asset can be obtained if such devices are outsourced to a non-serious (untrusted) third-party service provider.
9	This asset can be obtained if such information is sent from the tracking system to a listener device in clear text and is captured by an attacker.
10	This asset can be found physically or digitally, e.g., on papers, CDs, DVDs, backup media, a PC, communication networks or databases. The information can be accessed by unauthorized people if not stored properly and securely.

**Table 4:** Proposed security threat and risk countermeasures to be applied in IoT-based smart home environments.

Threat ID	Possible Mitigation Approaches
	Control access to the system using efficient biometric identifiers [49]
1	Implement a user awareness program to make users aware of social engineering
	Implement multi-factor authentication
	Avoid using insecure Wi-Fi, which gives hackers access to personal data
2	Set up a secure network before using a home automation application
	Be aware of stolen or lost devices
	Use a secure communication channel by utilizing a secure virtual private network (VPN)
3	Limit network traffic such that it is accessible only to authorized users
	Develop a security awareness training program for smart home inhabitants
	Use an intrusion detection system (IDS) / intrusion prevention system (IPS)
4	Use encryption mechanisms for security data transmission [50]
	Perform frequent data backups to keep copies of sensitive data
	Secure the physical locations of installed devices
5	Provide secure access to device configuration interfaces
	Replace the default usability configuration of installed devices
	Use commodity hardware and software to collect and examine network traffic [33,34]
6	Create backups of the working system’s configurations
	Always monitor system’s performance, looking for misbehavior incidents
	Apply a strong authentication mechanism such as fingerprint authentication [51]
7	Offer awareness and training programs regarding system security
	Ensure that system configurations are secure and performed by authentic people
	Restrict physical access to devices to only authentic people
8	Avoid infrastructure outsourcing to a third-party service provider
	Modify default device configurations to achieve a better security level
	Disable unnecessary location tracking services on mobile devices
9	Develop a good understanding of user privacy concerns
	Track system behavior to identify any suspicious privacy leakage
	Use only trusted and authentic networks (wired or wireless)
10	Share information carefully and in a restricted manner
	Use only trusted providers to receive technical support for hardware failures in smart home

## References

[1] King J., Awad A.I. (2016). A Distributed Security Mechanism for Resource-Constrained IoT Devices. Informatica (Slovenia).

[2] Ning H. (2013). Unit and Ubiquitous Internet of Things.

[3] Miller M. (2015). The Internet of Things: How Smart TVs, Smart Cars, Smart Homes, and Smart Cities are Changing the World.

[4] Al-Fuqaha A., Guizani M., Mohammadi M., Aledhari M., Ayyash M. (2015). Internet of Things: A Survey on Enabling Technologies, Protocols, and Applications. IEEE Commun. Surv. Tutor..

[5] Suryadevara N.K., Mukhopadhyay S.C. (2015). Smart Homes: Design, Implementation and Issues.

[6] Khan R., Khan S.U., Zaheer R., Khan S. Future Internet: The Internet of Things Architecture, Possible Applications and Key Challenges. Proceedings of the 10th International Conference on Frontiers of Information Technology.

[7] Fabi V., Spigliantini G., Corgnati S.P. (2017). Insights on Smart Home Concept and Occupants’ Interaction with Building Controls. Energy Procedia.

[8] Harper R. (2003). Inside the Smart Home: Ideas, Possibilities and Methods. Inside the Smart Home.

[9] Aarts E., Marzano S. (2003). The New Everyday: Views on Ambient Intelligence.

[10] Nunes R.J.C., Delgado J.C.M. An Internet Application for Home Automation. Proceedings of the 10th Mediterranean Electrotechnical Conference.

[11] Erdogan S.Z., Meghanathan N., Boumerdassi S., Chaki N., Nagamalai D. (2010). Mobility Monitoring by Using RSSI in Wireless Sensor Networks. Recent Trends in Networks and Communications, Proceedings of the International Conferences, NeCoM 2010, WiMoN 2010, WeST 2010, Chennai, India, 23–25 July 2010.

[12] Al-sumaiti A.S., Ahmed M.H., Salama M.M.A. (2014). Smart Home Activities: A Literature Review. Electr. Power Compon. Syst..

[13] Zupancic D., Cvetkovic B. (2014). Smart-home Energy Management in the Context of Occupants’ Activity. Informatica (Slovenia).

[14] Granzer W., Kastner W., Neugschwandtner G., Praus F. Security in Networked Building Automation Systems. Proceedings of the 2006 IEEE International Workshop on Factory Communication Systems.

[15] Al-Qutayri H.A., Jeedella J.S., Al-Qutayri M.A. (2010). IIntegrated Wireless Technologies for Smart Homes Applications. Smart Home Systems.

[16] Kyas O. (2013). How to Smart Home.

[17] De Silva L.C., Morikawa C., Petra I.M. (2012). State of the Art of Smart Homes. Eng. Appl. Artif. Intell..

[18] Acharjya D.P., Geetha M.K. (2017). Internet of Things: Novel Advances and Envisioned Applications.

[19] Shen B., Lin Y., Wang X. Research on Data Mining Models for the Internet of Things. Proceedings of the 2010 International Conference on Image Analysis and Signal Processing.

[20] Kang B., Liu F., Yun Z., Liang Y. Design of an Internet of Things-based Smart Home System. Proceedings of the 2011 2nd International Conference on Intelligent Control and Information Processing.

[21] Evans D. (2011). The Internet of Things: How the Next Evolution of the Internet is Changing Everything.

[22] Montano C., Lundmark M., Mähr W. Control vs. Convenience: Critical Factors of Smart Homes. Proceedings of the 2nd Scandinavian Student Interaction Design Research Conference.

[23] Bandyopadhyay S., Sengupta M., Maiti S., Dutta S., Özcan A., Zizka J., Nagamalai D. (2011). A Survey of Middleware for Internet of Things. Recent Trends in Wireless and Mobile Networks, Proceedings of the Third International Conferences, WiMo 2011 and CoNeCo 2011, Ankara, Turkey, 26–28 June 2011.

[24] Papadopoulos K., Zahariadis T., Leligou N., Voliotis S. Sensor Networks Security Issues in Augmented Home Environment. Proceedings of the 2008 IEEE International Symposium on Consumer Electronics.

[25] He J., Xiao Q., He P., Pathan M.S. (2017). An Adaptive Privacy Protection Method for Smart Home Environments Using Supervised Learning. Future Internet.

[26] Ricquebourg V., Menga D., Durand D., Marhic B., Delahoche L., Loge C. The Smart Home Concept: Our Immediate Future. Proceedings of the 2006 1st IEEE International Conference on E-Learning in Industrial Electronics.

[27] Chaqfeh M.A., Mohamed N. Challenges in Middleware Solutions for the Internet of Things. Proceedings of the 2012 International Conference on Collaboration Technologies and Systems (CTS).

[28] Yoo D.Y., Shin J.W., Choi J.Y. (2007). Home-network Security Model in Ubiquitous Environment. Proc. World Acad. Sci. Eng. Technol..

[29] Liu Y., Hu S., Ho T.Y. Vulnerability Assessment and Defense Technology for Smart Home Cybersecurity Considering Pricing Cyberattacks. Proceedings of the 2014 IEEE/ACM International Conference on Computer-Aided Design (ICCAD).

[30] Nixon P.A., Wagealla W., English C., Terzis S. (2005). Security, Privacy and Trust Issues in Smart Environments. Smart Environments.

[31] Schiefer M. Smart Home Definition and Security Threats. Proceedings of the 2015 Ninth International Conference on IT Security Incident Management IT Forensics.

[32] Can O., Sahingoz O.K. A Survey of Intrusion Detection Systems in Wireless Sensor Networks. Proceedings of the 2015 6th International Conference on Modeling, Simulation, and Applied Optimization (ICMSAO).

[33] Rubio-Loyola J., Sala D., Ali A.I. Maximizing Packet Loss Monitoring Accuracy for Reliable Trace Collections. Proceedings of the 16th IEEE Workshop on Local and Metropolitan Area Networks (LANMAN2008), Chij-Napoca.

[34] Rubio-Loyola J., Sala D., Ali A.I. Accurate Real-time Monitoring of Bottlenecks and Performance of Packet Trace Collection. Proceedings of the 33rd IEEE Conference on Local Computer Networks (LCN2008).

[35] Jacobsson A., Boldt M., Carlsson B. (2016). A Risk Analysis of a Smart Home Automation System. Future Gener. Comput. Syst..

[36] Jing Q., Vasilakos A.V., Wan J., Lu J., Qiu D. (2014). Security of the Internet of Things: Perspectives and Challenges. Wirel. Netw..

[37] Wu T., Zhao G. (2014). A Novel Risk Assessment Model for Privacy Security in Internet of Things. Wuhan Univ. J. Nat. Sci..

[38] Nurse J.R.C., Creese S., Roure D.D. (2017). Security Risk Assessment in Internet of Things Systems. IT Prof..

[39] Yang L., Yang S.H., Yao F. Safety and Security of Remote Monitoring and Control of Intelligent Home Environments. Proceedings of the 2006 IEEE International Conference on Systems, Man and Cybernetics.

[40] Mantoro T., Ayu M.A., Mahmod S.M.B. Securing the Authentication and Message Integrity for Smart Home using Smart Phone. Proceedings of the 2014 International Conference on Multimedia Computing and Systems (ICMCS).

[41] Tong J., Sun W., Wang L. An Information Flow Security Model for Home Area Network of Smart Grid. Proceedings of the 2013 IEEE International Conference on Cyber Technology in Automation, Control and Intelligent Systems.

[42] Caralli R.A., Stevens J.F., Young L.R., Wilson W.R. (2007). Introducing Octave Allegro: Improving the Information Security Risk Assessment Process.

[43] Caralli R., Stevens J., Young L., Wilson W. (2007). The OCTAVE Allegro Guidebook, v 1.0.

[44] Mouton F., Leenen L., Venter H. (2016). Social Engineering Attack Examples, Templates and Scenarios. Comput. Secur..

[45] Awad A.I., Hassanien A.E., Muda A.K., Choo Y.H., Abraham A.N., Srihari S. (2014). Impact of Some Biometric Modalities on Forensic Science. Computational Intelligence in Digital Forensics: Forensic Investigation and Applications.

[46] Okoh E., Awad A.I., Yin X., Ho K., Zeng D., Aickelin U., Zhou R., Wang H. (2015). Biometrics Applications in e-Health Security: A Preliminary Survey. Health Information Science.

[47] Awad A.I., Balas V.E., Jain L.C., Kovačević B. (2016). Fast Fingerprint Orientation Field Estimation Incorporating General Purpose GPU. Soft Computing Applications.

[48] Stallings W., Brown L. (2014). Computer Security: Principles and Practice.

[49] Awad A.I., Baba K. Evaluation of a Fingerprint Identification Algorithm with SIFT Features. Proceedings of the 3rd 2012 IIAI International Conference on Advanced Applied Informatics.

[50] Elfatah A.F.A., Tarrad I.F., Awad A.I., Hamed H.F.A. Optimized Hardware Implementation of the Advanced Encryption Standard Algorithm. Proceedings of the 8th International Conference on Computer Engineering Systems (ICCES).

[51] Awad A.I., Baba K. (2011). Fingerprint Singularity Detection: A Comparative Study. Software Engineering and Computer Systems.

[52] Luo T., Hao H., Du W., Wang Y., Yin H. (2011). Attacks on WebView in the Android System. Proceedings of the 27th Annual Computer Security Applications Conference.

[53] Krupp B., Sridhar N., Zhao W. (2017). SPE: Security and Privacy Enhancement Framework for Mobile Devices. IEEE Trans. Dependable Secur. Comput..

[54] Bako A. (2016). Internet of Things Based Smart Homes: Security Risk Assessment and Recommendations. Master’s Thesis.

[55] Das S., Chakravarthi V.S., Shirur Y.J.M., Prasad R. (2013). Technology for Smart Home. Proceedings of the International Conference on VLSI, Communication, Advanced Devices, Signals & Systems and Networking (VCASAN-2013).

[56] Khan S.H., Akbar M.A., Shahzad F., Farooq M., Khan Z. (2015). Secure Biometric Template Generation for Multi-factor Authentication. Pattern Recognit..

[57] Zadran H. Amazing Architecture, 2017. http://amazingarchitecture.net/2017/05/19/elegant-home-plan-design-ideas/.

[58] Pirbhulal S., Zhang H., E Alahi M.E., Ghayvat H., Mukhopadhyay S.C., Zhang Y.T., Wu W. (2016). A Novel Secure IoT-based Smart Home Automation System using a Wireless Sensor Network. Sensors.

[59] Suárez-Albela M., Fernández-Caramés T.M., Fraga-Lamas P., Castedo L. (2017). A Practical Evaluation of a High-Security Energy-Efficient Gateway for IoT Fog Computing Applications. Sensors.

[60] Moosavi S.R., Gia T.N., Rahmani A.M., Nigussie E., Virtanen S., Isoaho J., Tenhunen H. (2015). SEA: A Secure and Efficient Authentication and Authorization Architecture for IoT-Based Healthcare Using Smart Gateways. Procedia Comput. Sci..

[61] Gajewski M., Batalla J.M., Mastorakis G., Mavromoustakis C.X. (2017). A Distributed IDS Architecture Model for Smart Home Systems. Clust. Comput..

[62] Fathy A., Tarrad I.F., Hamed H.F.A., Awad A.I., Hassanien A.E., Salem A.M., Ramadan R., Kim T. (2012). Advanced Encryption Standard Algorithm: Issues and Implementation Aspects. Advanced Machine Learning Technologies and Applications.

[63] Awad A.I. (2013). Fingerprint Local Invariant Feature Extraction on GPU with CUDA. Informatica (Slovenia).

[64] Egawa S., Awad A.I., Baba K., Benlamri R. (2012). Evaluation of Acceleration Algorithm for Biometric Identification. Networked Digital Technologies.

[65] Bilal M., Kang S.G. (2017). An Authentication Protocol for Future Sensor Networks. Sensors.

